# Synthesis and Biological Evaluation of Dantrolene‐Like Hydrazide and Hydrazone Analogues as Multitarget Agents for Neurodegenerative Diseases

**DOI:** 10.1002/cmdc.202100209

**Published:** 2021-06-22

**Authors:** Isabella Bolognino, Nicola Giangregorio, Annamaria Tonazzi, Antón L. Martínez, Cosimo D. Altomare, María I. Loza, Sara Sablone, Saverio Cellamare, Marco Catto

**Affiliations:** ^1^ Department of Pharmacy-Pharmaceutical Sciences University of Bari Aldo Moro Via E. Orabona 4 70125 Bari Italy; ^2^ Institute of Biomembranes Bioenergetics and Molecular Biotechnologies (IBIOM) National Research Council (CNR) Via Amendola 122/O 70126 Bari Italy; ^3^ BioFarma Research Group Center for Research in Molecular Medicine and Chronic Diseases (CiMUS) University of Santiago de Compostela Av. Barcelona, Campus Vida 15782 Santiago de Compostela Spain; ^4^ Section of Legal Medicine Interdisciplinary Department of Medicine Bari Policlinico Hospital University of Bari Aldo Moro Piazza Giulio Cesare 11 70124 Bari Italy; ^5^ Department of Engineering and Applied Sciences University of Bergamo Viale G. Marconi 5 24044 Dalmine Italy

**Keywords:** dantrolene analogues, hydrazide and hydrazone derivatives, multitarget activity, carnitine/acylcarnitine carrier, Alzheimer's disease

## Abstract

Dantrolene, a drug used for the management of malignant hyperthermia, had been recently evaluated for prospective repurposing as multitarget agent for neurodegenerative syndromes, including Alzheimer's disease (AD). Herein, twenty‐one dantrolene‐like hydrazide and hydrazone analogues were synthesized with the aim of exploring structure‐activity relationships (SARs) for the inhibition of human monoamine oxidases (MAOs) and acetylcholinesterase (AChE), two well‐established target enzymes for anti‐AD drugs. With few exceptions, the newly synthesized compounds exhibited selectivity toward MAO B over either MAO A or AChE, with the secondary aldimine **9** and phenylhydrazone **20** attaining IC_50_ values of 0.68 and 0.81 μM, respectively. While no general SAR trend was observed with lipophilicity descriptors, a molecular simplification strategy allowed the main pharmacophore features to be identified, which are responsible for the inhibitory activity toward MAO B. Finally, further in vitro investigations revealed cell protection from oxidative insult and activation of carnitine/acylcarnitine carrier as concomitant biological activities responsible for neuroprotection by hits **9** and **20** and other promising compounds in the examined series.

## Graphical abstract

**Simpler is better**: Structural modifications of the chemical structure of dantrolene, previously disclosed as a repositionable drug with potential for Alzheimer's disease, allowed preparing new small molecules with multitarget activity profile. Compared to dantrolene, molecular simplification resulted in less toxic and more soluble compounds. Some of them had higher potency as selective MAO B inhibitors, and were effective in cell protection from oxidative insult and in the activation of CAC carnitine carrier.

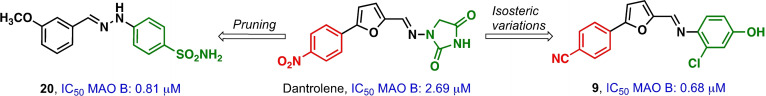

## Introduction

**Figure 1:**
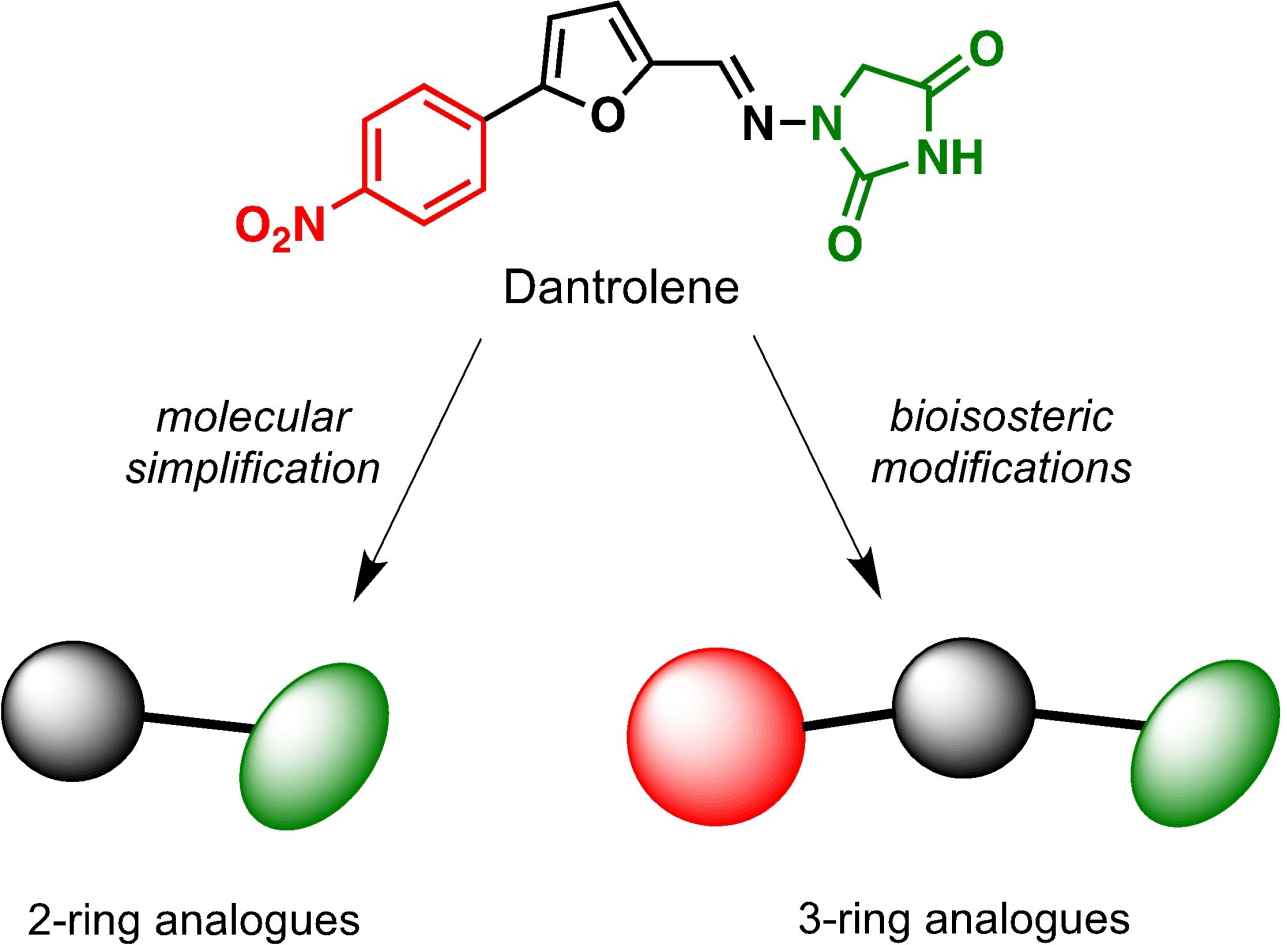
General strategy for the synthesis of hydrazide/hydrazone derivatives.

Dantrolene (DAN; Figure 1) is a drug specifically used in the management of malignant hyperthermia, a life‐threatening pathology with fatal course. In a recent work, we disclosed new biological activities exerted by DAN, namely inhibition of monoamine oxidase (MAO) B human enzyme with *K*_i_ value in the low micromolar range, acetylcholinesterase (AChE), and aggregation of beta amyloid‐40 and hexapeptide tau protein sequence PHF6, i. e. two probes of amyloid aggregation in Alzheimer's disease (AD) brain.^[1]^ It is well known the crucial role of MAO isoforms A and B as metabolizing enzymes in modulating the concentration of neurotransmitters, mostly in some severe and chronic neurodegenerative pathologies. This established reputation is strictly related to the substrate and tissue specificity of both isoforms: MAO A selective inhibitors are clinically administered as antidepressants,^[2]^ while MAO B selective inhibition is commonly used for the treatment of the early symptoms of Parkinson's disease.^[3]^ A new outcome of that study was the discovery of the activation by DAN of the carnitine/acylcarnitine carrier (CAC), with EC_50_ of 9.3 μM for the purified recombinant wild type (WT) protein. This transporter acts through reductive activation and is involved in trafficking of acyl groups into the mitochondria, carried by l‐carnitine. Treated with DAN, this transport system facilitates, under oxidative stress (OS) conditions, the restoring of ATP production and thus cell vitality, but also the export of endogenous acetyl‐l‐carnitine from mitochondria, with consequent neuroprotective effects.

The above promising results prompted us to synthesize a number of novel DAN analogues, with the aim of optimizing the inhibitory activity against MAO B and AChE, ultimately improving their pleiotropic pharmacological potential in the treatment of AD and related neurodegenerative syndromes. In this work we investigated in particular: i) the bioisosteric replacement of the NO_2_ group, which may be toxicophore and precursor for the production of reactive oxygen species (ROS), with CN group; ii) the improvement of the aqueous solubility of DAN by replacing the hydantoin moiety with other (hetero)cyclic moieties bearing protonatable nitrogen(s); iii) the effect of molecular simplification of the three‐ring scaffold in DAN, for detecting the minimal pharmacophoric features responsible for the dual activity on MAO A/B and CAC (Figure 1). SARs were investigated as thoroughly as possible, even considering the effect of lipophilicity on the enzymes’ inhibition potency.

**Figure 2:**
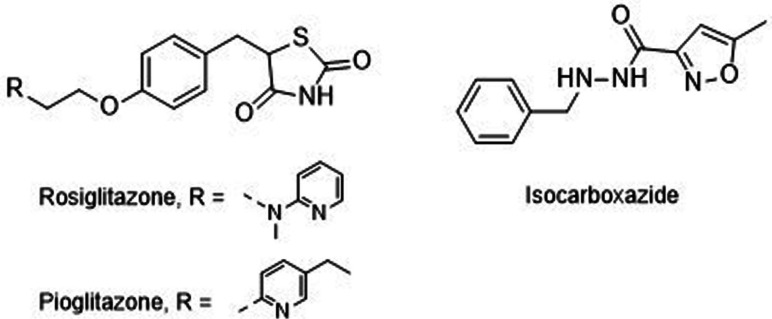
MAO inhibitors structurally related to dantrolene.

Some of the prepared compounds retained the molecular motif of hydantoin, present in DAN and, as thiohydantoin, in the known hypoglycemic drugs rosiglitazone and pioglitazone (Figure 2), which also behave as moderate MAO inhibitors. In turn, the molecular pruning gave simple hydrazone and hydrazide derivatives (2‐rings series, compounds **14**–**21**) whose structural pattern could be related to that of isocarboxazide (Figure 2), an early irreversible MAO inhibitor.

## Results and Discussion

### Chemistry

**Scheme 1:**
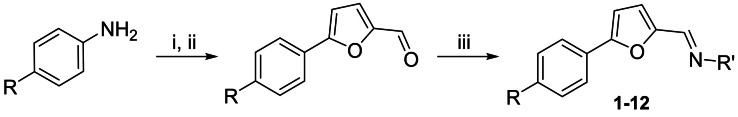
Reagents and conditions: (i) HCl, 0 °C, NaNO_2_, rt, 30 min; (ii) 2‐furaldehyde, CuCl_2_, acetone, rt; (iii) amine or hydrazine or hydrazide, DMF/water, HCl (cat.), rt, 24 h.

**Scheme 2:**
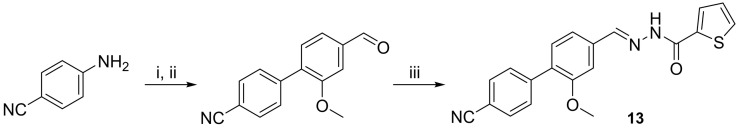
Reagents and conditions: (i) HCl, 0 °C, NaNO_2_, rt, 30 min; (ii) 3‐methoxybenzaldehyde, CuCl_2_, acetone, rt; (iii) 2‐thiophenecarboxylic acid hydrazide, DMF/water, HCl (cat.), rt, 24 h.

**Scheme 3:**
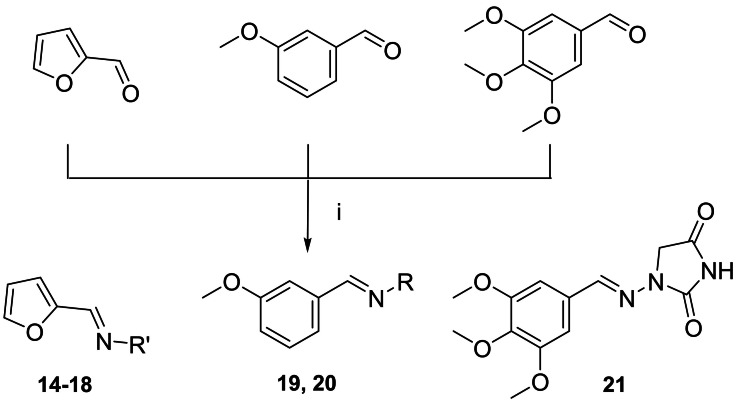
Reagents and conditions: (i) hydrazine or hydrazide, acetone/water, HCl (cat.), rt, 24 h.

The synthetic pathways chosen for the preparation of the designed compounds are those explored by Snyder and coworkers with slight modifications.^[4]^ The chemical scaffolds were selected to investigate a range of molecular diversity around the structure of DAN, in terms of variation of stereo‐electronic and hydrophobic properties. The general strategy of structural modifications is shown in Figure 1, whereas the syntheses of compounds are shown in Schemes 1–3. Condensations from suitable aldehydes and amino/hydrazide derivatives were performed in DMF/water or acetone/water mixtures, with acidic catalysis and agitation at room temperature. Final compounds were obtained in moderate (20–50 %) to good (70 %) yields, following a simple workup including filtration and purification through either crystallization or column chromatography.

### Inhibition of MAOs and AChE

The results of in vitro inhibition tests on human MAOs and AChE of the newly synthesized DAN analogues are summarized in Tables 1 and 2, along with the inhibition data of pargyline and galantamine used as positive controls for MAO (B‐selective) and AChE, respectively. Inhibition assays were also performed on human butyrylcholinesterase (BChE), where all compounds resulted inactive or poorly active (data not shown). IC_50_ values were calculated for compounds displaying >60 % inhibition in one‐point (10 μM) concentration assay.

**Table 1:** Inhibition of MAOs and AChE (compounds **1**–**13**).


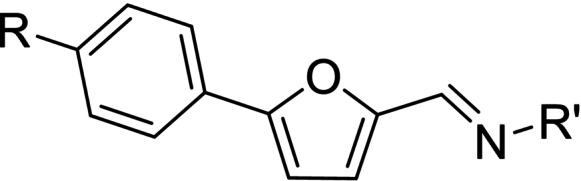							

Entry	R	R’	IC_50_ [μM] or *% inhibition at 10* *μM*			Clog P^[a]^	log *k*’^[b]^
			*h*MAO A	*h*MAO B	*h*AChE		
							
**DAN**	NO_2_	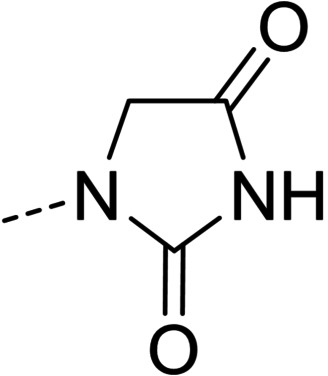	14.0±1.0^c^	2.69±0.44^c^	4.19±0.73^c^	1.63	0.115
**7**	CN		*40 % ±5*	*50 % ±3*	*28 %±4*	1.32	–
							
**1**	NO_2_	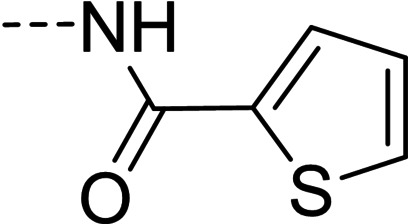	*33 %±2*	3.67±0.92	*40 %±4*	4.18	0.947
**4**	CN		1.46±0.21	2.63±0.42	*51 %±3*	3.12	–
							
**2**	NO_2_	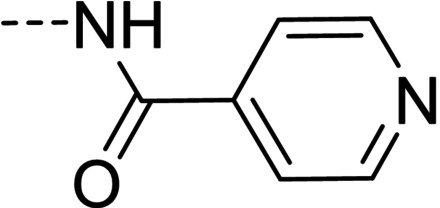	7.89±1.10	3.56±0.36	*30 %±1*	2.63	0.522
**5**	CN		6.62±0.71	12.6±0.9	*27 %±2*	2.32	0.312
							
**3**	NO_2_	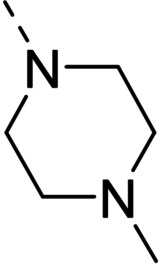	5.61±1.11	3.15±0.03	*44 %±2*	2.58	0.752
							
**6**	CN	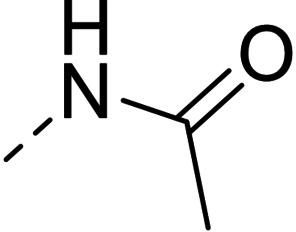	4.25±0.45	2.65±0.11	*38 %±4*	1.88	0.236
							
**8**	CN	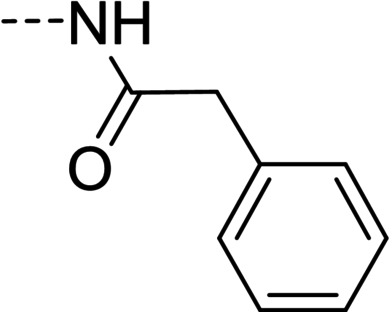	5.51±0.54	3.61±0.04	*16 %±1*	3.43	0.803
							
**9**	CN	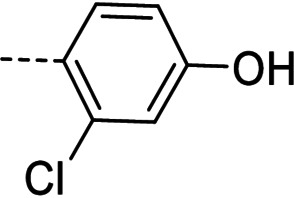	3.46±0.25	0.68±0.05	*30 %±0.4*	3.95	0.782
							
**10**	CN	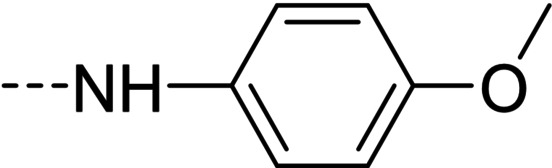	*47 %±4*	*29 %±4*	*55±1*	4.14	–
							
**11**	CN	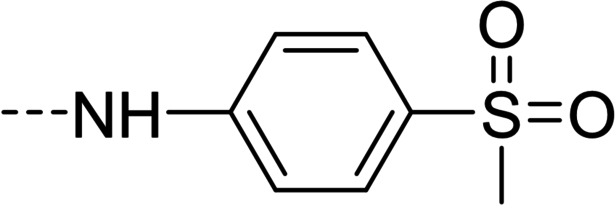	*11 %±4*	*28 %±3*	*39 %±4*	3.15	–
							
**12**	CN	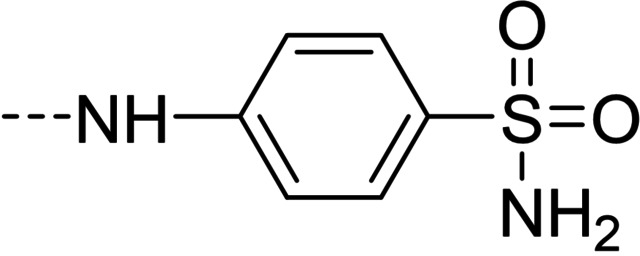	10.0±1.9	*28 %±4.0*	1.67±0.19	2.58	0.466
					
**13**			*29 %±4*	2.32±0.43	*35 %±5*	3.19	0.549
**Pargyline**			10.9±0.6	2.69±0.48	–	–	–
**Galantamine**			–	–	0.72±0.15	–	–

[a] ChemDraw software 15.0. [b] Logarithm of the capacity factor *k*’ (defined as (t_R_‐t_0_)/t_0_, where t_R_ is the retention time and t_0_ the dead time) measured by RP‐HPLC using MeOH/phosphate buffer pH 7.4 gradient from 80 : 20 to 60 : 40 v/v. [c] Data taken from Ref. [1].

**Table 2:** Inhibition of MAOs and AChE (compounds **14**–**21**).


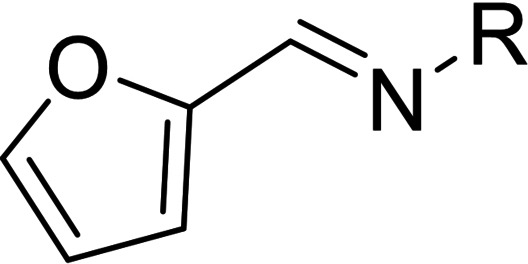						

Entry	R	IC_50_ [μM] or *% inhibition at 10* *μM*			Clog P^[a]^	log *k*’^[b]^
		*h*MAO A	*h*MAO B	*h*AChE		
						
**14**	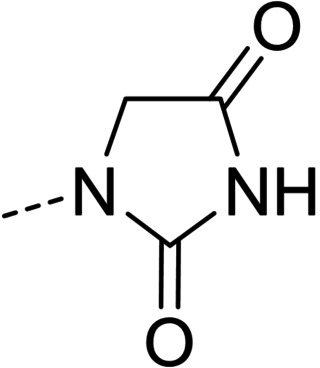	*11 %±3*	no inhibition	9.06±0.45	−0.21	−1.018
						
**15**	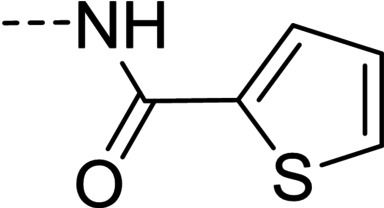	*16 %±4*	*13 %±3*	7.18±0.07	1.59	−0.010
						
**16**	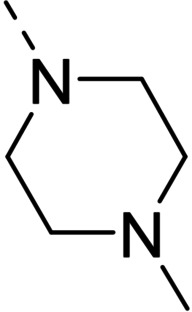	*18 %±3*	*13 %±4*	*21 %±5*	1.27	–
						
**17**	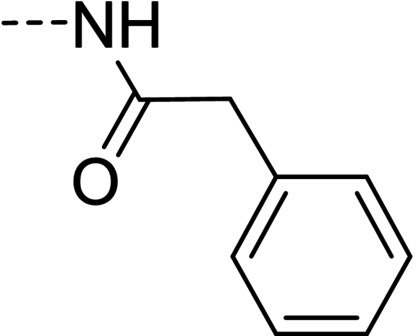	*22 %±5*	*22 %±4*	*52 %±4*	1.89	–
						
**18**	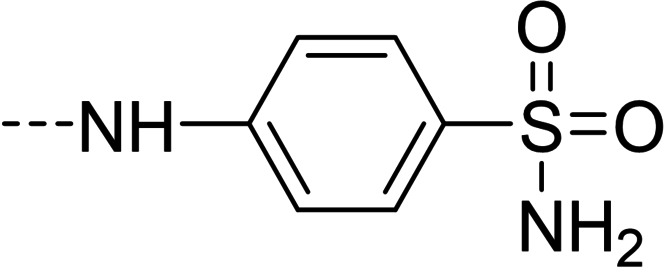	*25 %±5*	*21 %±3*	*27 %±5*	1.03	–


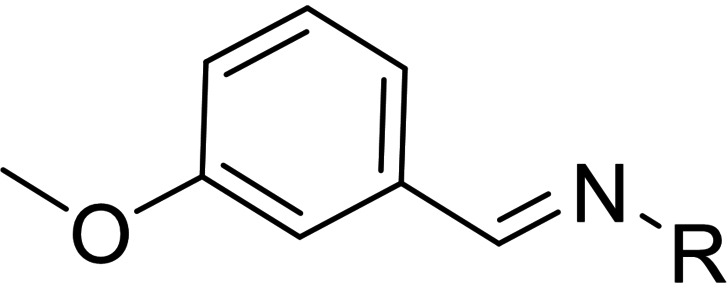						

**19**	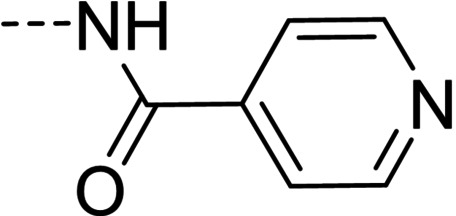	*19 %±5*	12.2±1.5	11.2±1.6	1.69	0.129
						
**20**	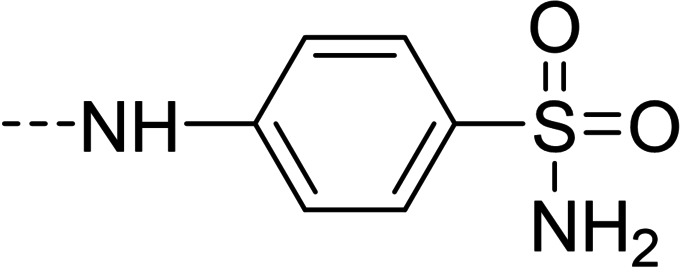	*42 %±2*	0.81±0.04	10.2±1.6	2.09	0.307
					
**21**		*8 %±2*	*18 %±5*	*28 %±4*	−0.08	–

[a], [b] see Footnotes of Table 1.

Compared with previously obtained results of DAN,^[1]^ its CN congener **7** showed a significant loss of inhibition potency toward all three enzymes (approximately a four‐fold decrease for MAO B). On the contrary, the thiophene hydrazides **1** and **4** proved to be equipotent with DAN for MAO B inhibition, with a noteworthy increase of MAO A activity and loss of MAO B/A selectivity for the CN congener **4**. The same modification in compounds **2** and **5** resulted in almost overlapping activities, even with a decrease of MAO B potency.

Compound **3**, which along with **1** and **2** maintains the 4‐nitrophenyl substituent, was prepared with the aim of introducing a strong basic moiety likely able to improve the affinity to cholinesterases. Unfortunately, it resulted a poor AChE inhibitor, while retaining fair MAO inhibition, although unselective, in the low micromolar range. The same activity profile was featured by the hydrazide **6** and **8**, where the structural simplification of the hydantoin ring of the close analogue **7** to acethydrazide (**6**) and phenylacethydrazide (**8**) allowed the restoration of MAO inhibitory activity.

The introduction of terminal moieties with larger structural diversity, as in case of compounds **9**–**13**, produced contrasting results. Concerning MAO B inhibition, we obtained an interesting submicromolar value of IC_50_ for phenol derivative **9**, which turned out as the most potent MAO B inhibitor of this series, also displaying 5‐fold selectivity over MAO A.

**Figure 3:**
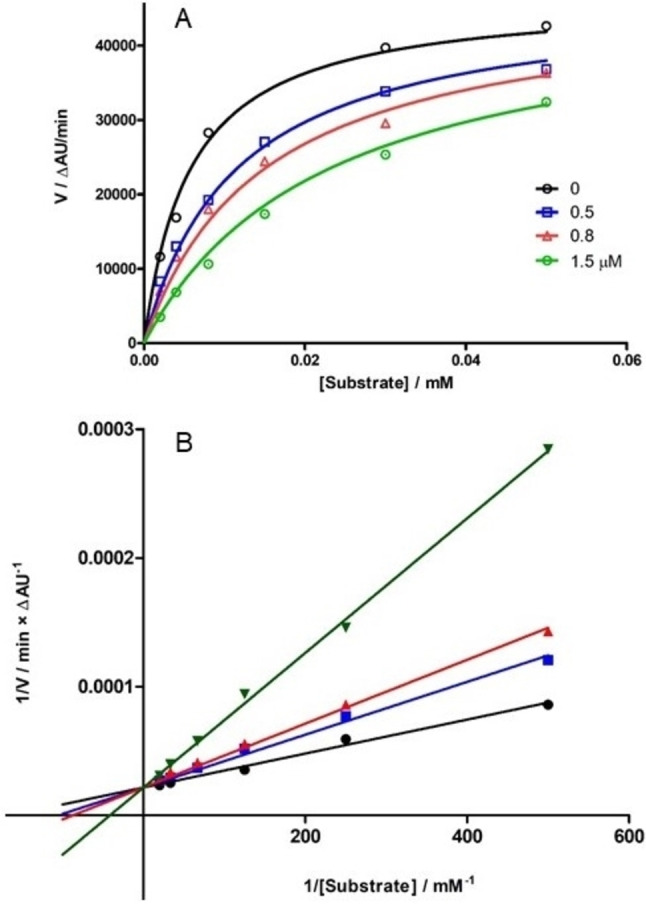
Michaelis‐Menten plot (A) and Lineweaver‐Burk linearization (B) of inhibition kinetics of *h*MAO B with compound **9**. Image is representative of a single experiment.

Inhibition kinetics assessed for **9** a competitive mechanism, with *K*_i_ equal to 0.50±0.06 μM (Figure 3). Taking into account the possible hydrolytic degradation of imine (see stability studies below), inhibition kinetics were determined without preincubation with substrate, as usually done for MAO inhibition experiments.

The derivatization of furaldehyde bridge as 4‐substituted arylhydrazones (compounds **10**–**12**) resulted in a decrease of activity towards MAO isoforms. Nevertheless, sulfonamide derivative **12** scored an unexpectedly remarkable inhibitory activity towards AChE with an IC_50_ value of 1.67 μM.

While compound **4** was the most active inhibitor of MAO A (IC_50_ 1.46 μM), the replacement of the central furan ring with a larger and sterically hindered ring such as methoxybenzene in **13**, resulted in a strong decrease of this inhibitory activity, while retaining a similar low value of IC_50_ for MAO B. This potency reversal represents an interesting example of isoform selectivity, but seems also contrasting with previous results,^[5]^ assessing a preferential MAO A affinity for molecules bearing bulky bridging substituents.

A number of structurally simpler derivatives, based on two‐ring scaffolds, were also synthesized with the aim of gaining information on the minimal pharmacophore features^[6]^ of the compounds under examination. The effects of such a molecular pruning on target interactions are shown in Table 2. Removal of nitrophenyl group (compare **14** vs. DAN and **7**, **15** vs. **1** and **4**, **16** vs. **3**, **17** vs. **8**, **18** vs. **12**) resulted in a marked loss of activity towards MAOs, while only in few cases AChE inhibition was sparingly retained, particularly in the case of isonicotinic acid derivative **19**, analogue of **2** and **5**. It is worthy of note that sulfonamide **18** lost the appreciable AChE inhibitory activity of its analogue **12**, likely because of the lack of favorable hydrophobic/aromatic interactions by the nitrophenyl moiety in the enzyme binding site. This hypothesis is strengthened by the result of compound **20**, which can be considered as a superior homologue of **18**. The increase of lipophilicity, along with the introduction of an electron‐donating substituent, through the replacement of furan with the methoxybenzene ring determined not only the restoring of the activity towards *h*AChE but also, in line with expectations, the restoring of the affinity towards MAO targets. Particularly in MAO B inhibition (IC_50_=0.81 μM), sulfonamide **20** resulted as a potent and B/A selective candidate within the entire molecular set herein presented.

Lastly the introduction of a sterically hindered ring such as 3,4,5‐trimetoxybenzene (**21**) caused a loss of activity (8–28 % inhibition) towards both MAOs and AChE at the highest concentration tested (10 μM).

**Figure 4:**
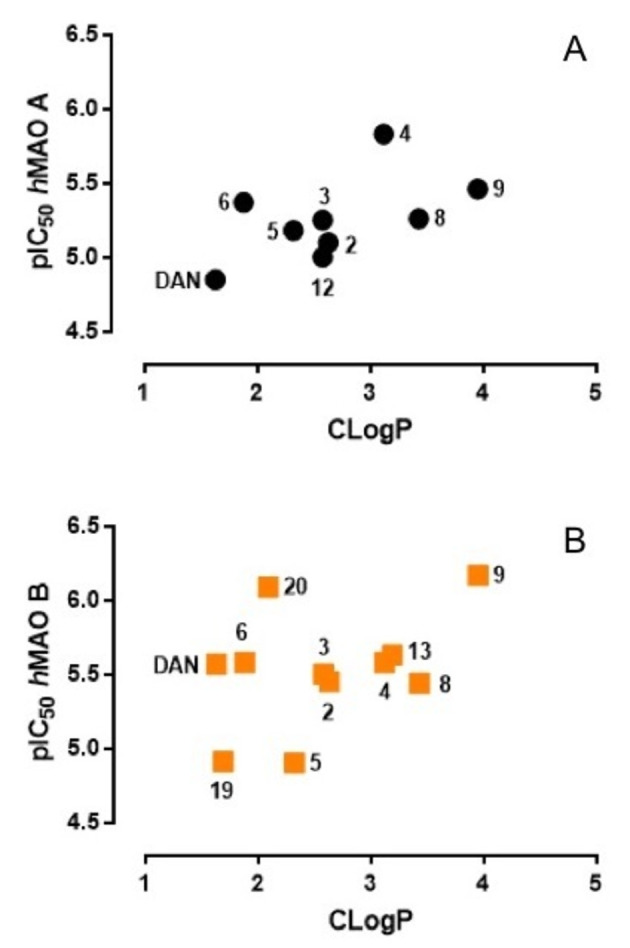
Plots of pIC_50_ values determined toward MAO A (A) and B (B) *versus* calculated Clog P values (ChemDraw); only compounds with finite IC_50_ values are shown.

To investigate a possible correlation between MAO inhibitory potency and lipophilicity,^[7]^ we calculated log P with three different programs (ChemDraw 15.0; ALOG PS 2.1; ChemSketch 2017, see Table S1 in Supporting Information) and compared the calculated values with experimental lipophilicity indexes as assessed by reversed‐phase (RP) HPLC in isocratic conditions for the majority of compounds. The log of capacity factors (log *k*’) measured by RP‐HPLC using a mixture of methanol/PBS (60 : 40, v/v) as the mobile phase are reported in Tables 1 and 2, along with Clog P values calculated with ChemDraw. These calculated lipophilicity descriptors correlated with the experimental log *k*’ values (n=13, *r* ^2^=0.901) better than the other log Ps calculated by ALOG PS and ChemSketch computational tools (*r* ^2^ equal 0.705 and 0.568, respectively). As shown by the scatter plots in Figure 4 and Figure S1 in Supporting Information, there is no evident correlation trend between MAO A/B inhibition data and lipophilicity calculated by the expert system implemented in ChemDraw for compounds achieving finite IC_50_ values.

While the graphical analysis proved the absence of a general correlation trend, the experimental log *k’* values (Tables 1 and 2), albeit limited to just over half of the compounds studied, suggest a certain effect of lipophilicity on the inhibition potency. Indeed, within the physicochemical property space explored, the strongest MAO A/B inhibitors possess higher lipophilicity (i. e., with values comprised in the 0.0–1.0 range), whereas the most active AChE inhibitors scored log *k’* values close to or lower than 0.

### Cell‐based assay of neuroprotection

In order to get further information about the potential neuroprotective effects exerted by the most active compound of the molecular series (phenol derivative **9**) toward *h*MAO B, we tested it in a 2′,7′‐dichlorofluorescein diacetate (DCF‐DA) fluorescence‐based assay measuring the production of ROS induced by dopamine in cultured SH‐SY5Y cells.

**Figure 5:**
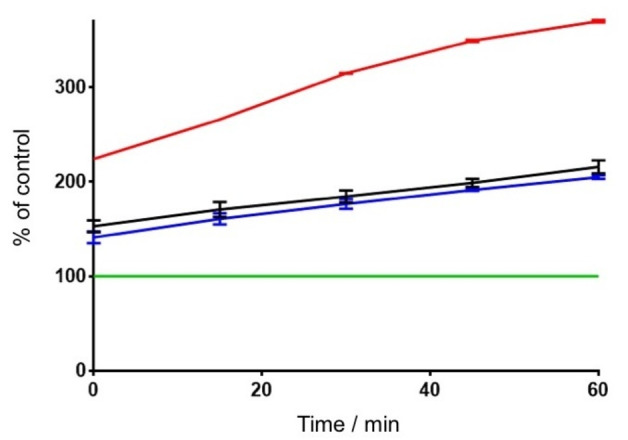
Neuroprotection of SH‐SY5Y cells from oxidative insult; DCF‐DA assay. Green line, control cells; purple line, 10 nM dopamine; blue line, 10 nM dopamine+10 nM phenelzine; black line, 10 nM dopamine+10 nM **9**. The data represent mean±SD of three independent experiments.

The immortalized neuroblastoma cells are a widely used model for neuroprotection assays, while dopamine at a concentration of 10 nM acts as an inducer of oxidative stress,[^8^, ^9^] being metabolized by MAOs to form hydrogen peroxide. Once hydrolyzed and oxidized, DCF acts as the fluorescing probe of the oxidative stress (OS) state of cells. In the presence of an antioxidant, or even of a MAO inhibitor, ROS burden is lowered and DCF fluorescence decreased. Figure 5 shows that **9** was effective in reducing ROS oxidation of DCF. Its activity is superimposable to that of phenelzine, a nonselective MAO inhibitor used as a positive control in this test.^[10]^ The same experiment performed on dantrolene in our previous work gave similar results.^[1]^

### Hydrolytic stability in buffered solution

**Figure 6:**
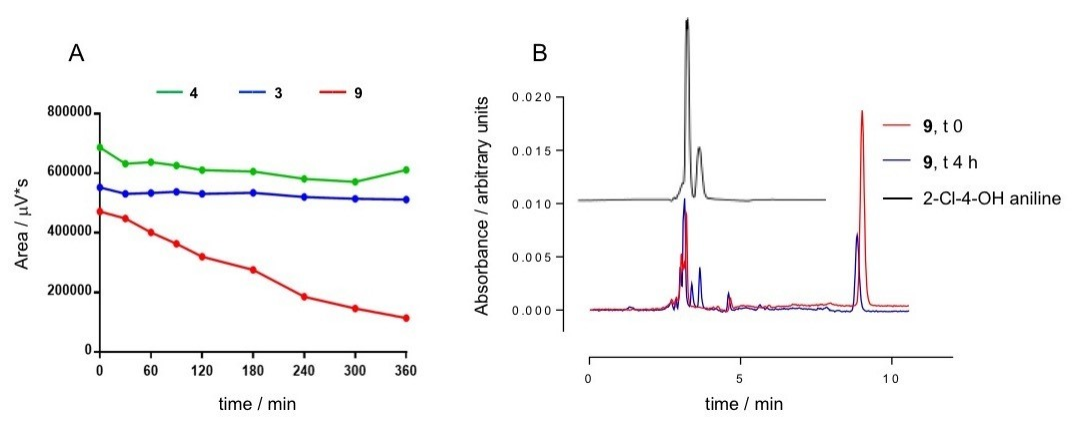
(A) time‐resolved stability of compounds **3**, **4** and **9** at the concentration of 20 μM in PBS at 37 °C. Data are representative of three independent experiments and values expressed as mean. (B) overlapping chromatographic peaks of **9** at t 0 and t 4 h and chromatogram of 2‐Cl‐4‐OH aniline.

The hydrolytic stability of some representative imino/hydrazone/hydrazide derivatives was determined in buffered aqueous media. The stability studies were carried out on compounds **3**, **4** and **9**, the most active MAO B inhibitors of the 3‐rings scaffold series, at a single concentration of 20 μM in 10 mM phosphate buffered saline (PBS) at physiological pH of 7.4, at 37 °C and for 6 h incubation time. The degradation profiles in Figure 6A confirmed a substantial stability for **3** and **4**, while the imine **9** resulted, at the end of the 6 h of incubation, in a degradation percentage of 76 %. This result is in line with the nature of the functional group of this molecule: in fact, **9** is the only Schiff base within the synthesized series and therefore by itself less stable than the other hydrazone analogues. The hydrolytic degradation of **9** in its starting reagents was confirmed by the comparison of the chromatograms of **9** and 2‐chloro‐4‐hydroxyaniline, obtained in the same chromatographic conditions (Figure 6B).

### Activation of carnitine/acylcarnitine carrier (CAC) transport

**Figure 7:**
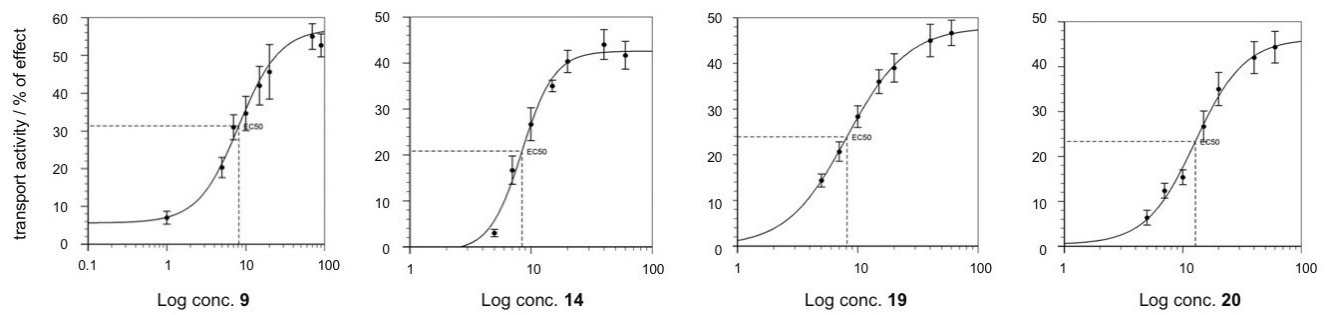
Dose‐response curves of activation of CAC (purified recombinant WT protein). The antiport rate was measured by adding 0.1 mM [^3^H]‐carnitine to proteoliposomes containing 15 mM internal carnitine and stopped after 30 min by the specific inhibitor N‐ethylmaleimide (NEM). Compounds at increasing concentrations were added 2 min before the transport assay. Values are mean±SD from three independent experiments.

CAC (SLC25 A20) is essential for the transport into mitochondria of acyl moieties as acylcarnitines, where they are processed by β‐oxidation pathway. The protein contains six cysteine residues, but two of them, namely C136 and C155, based on the redox state of the protein, are crucial for the regular function of the carrier.[^11^, ^12^] In fact, the transporter is active when the two cysteines are in reduced form, while it is inhibited when a C136‐C155 disulfide bridge is formed in conditions of OS. One or both cysteines represent also specific targets for various chemical and physiological thiol reducing agents,[^13^, ^14^, ^15^, ^16^, ^17^] allowing to modulate the transport activity of the carrier. We demonstrated that DAN led to a significant recovery of the CAC transport activity of the oxidized protein.^[1]^ Herein, we also investigated whether some newly synthesized derivatives, namely the most active MAO B inhibitors **9** and **20**, the DAN homologue **14**, and the dual MAO B/AChE inhibitor **19**, are effective in activating CAC. To calculate the EC_50_ values from dose‐response curves, that is, the concentration which increases the transport activity of the carrier by 50 % compared to the control, a wide concentration range (1–100 μM) was tested (Figure 7). The EC_50_ values measured after 30 min of incubation were 8.2±2.8 μM (comp. **9**), 8.4±1.6 μM (**14**), 8.2±0.57 μM (**19**), 13±1.8 μM (**20**), whereas the whole activation of the WT protein was observed at concentrations close to 100 μM for **8**, **19** and **20**, and 50 μM for **14**.

The tested molecules were able to improve the transport activity of CAC compared to the control and at least three of them (**9**, **14** and **19**) showed EC_50_ lower than that previously calculated for DAN (9.3 μM) after the same exposure duration (30 min), highlighting similar pharmacological effect. On the contrary, the efficacy of these compounds, i. e., the power of the molecules to achieve maximum effect, is about 5 times lower than DAN.^[1]^

**Figure 8:**
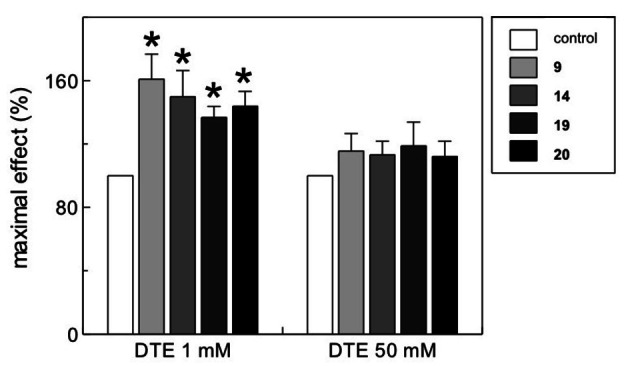
Effects of DAN analogues on the recombinant WT CAC protein. The proteoliposomes were prepared in two different reducing conditions, adding to the reconstitution mixture 1 or 50 mM DTE. Thus, the antiport rate was measured incubating the reconstituted protein with test molecules at 10 μM final concentration together with 0.1 mM [^3^H]‐carnitine and then stopping the transport activity after 30 min by NEM. The values are means±SD from three independent experiments, significantly different from the controls, as calculated from Student's *t*‐test analysis (* *p*<0.01).

To demonstrate that the action of DAN analogues was exerted on the cysteine residues of the CAC, 1 or 50 mM dithioerythritol (DTE), a strong reducing agent, was added to the reconstitution mixture (see Experimental section) in order to mimic the protein at different states of oxidation. The bar plot in Figure 8 shows that the tested molecules enabled the protein to recover a significant transport activity, compared to the control, when the protein is more oxidized, i. e., in the presence of 1 mM DTE.

## Conclusion

From our previous investigation of the multitarget activity exerted by DAN,^[1]^ we evidenced the potential of repurposing of this orphan drug toward AD‐related targets, but also its intrinsic limitations, particularly its low aqueous solubility, that may discourage further pharmacological evaluation. The DAN‐like molecules herein reported were designed and synthesized with the aim of extending the knowledge of the SARs and improving the pharmaceutical potential of this class of compounds. In particular, our efforts were focused on modifying the structure of DAN for increasing the aqueous solubility and replacing the potentially toxicophore nitro group, as well as following an approach of molecular simplification. The in vitro screening proved that some of the newly investigated analogues improved the MAO inhibitory potency, mostly for the 3‐ring series (compounds **1**–**13**), although with low isoenzyme selectivity. The phenol derivative **9** emerged as an outstanding, reversible MAO B inhibitor (*K*_i_ 0.50 μM) with fair B/A selectivity. The drop of MAO A activity of compound **13**, compared to that of its close congener **4**, is a matter of evidence that would deserve further investigations, considering the high B/A selectivity obtained with this homologation. Among the 2‐ring series, only the sulfonamide hydrazine **20** resulted in a strong and selective MAO B inhibitor (IC_50_ 0.81 μM). As far as the AChE inhibition is concerned, appreciable inhibition values were obtained only sparsely, in line with results recently published for a related class of DAN analogues.^[18]^ While only sulfonamide **12** resulted in good AChE inhibition, the best selectivity was achieved with compounds **14** and **15** resulting from the molecular simplification study. The limited molecular size of both 2‐ring and 3‐ring scaffold hampered a significant inhibition of BChE.

The new compounds confirmed a good stability in buffered conditions, with the obvious exception of imine **9**, and a safe cellular activity in contrasting ROS cytotoxicity from oxidative degradation of dopamine. Finally, some tested compounds confirmed the activation of the mitochondrial carnitine trafficking mediated by CAC, thus giving further evidence to the multitarget profile of this class of molecules. The evaluation of possible activity on ryanodine receptors will be a major concern to be faced in the near future, in order to achieve a more complete activity profile of these DAN analogues.

## Experimental Section

### Chemistry

Chemicals, solvents and reagents used for the syntheses were purchased from Sigma‐Aldrich (Milan, Italy) or Alfa Aesar (Haverhill, Massachusetts, USA) and used without any further purification. The purity of all intermediates, checked by ^1^H NMR and HPLC, was always higher than >95 %. Column chromatography was performed using Merck silica gel 60 (0.063–0.200 mm, 70–230 mesh). All reactions were routinely checked by TLC using Merck Kieselgel 60 F254 aluminum plates and visualized by UV light. Nuclear magnetic resonance spectra were recorded on a Varian Mercury 300 instrument (at 300 MHz) or on Agilent Technologies 500 apparatus (at 500 MHz) at ambient temperature in the specified deuterated solvent. Chemical shifts (*δ*) are quoted in parts per million (ppm) and are referenced to the residual solvent peak. The coupling constants *J* are given in Hertz (Hz). The following abbreviations were used: s (singlet), d (doublet), dd (doublet of doublet), t (triplet), q (quadruplet), qn (quintuplet), m (multiplet), br s (broad signal); signals due to OH and NH protons were located by deuterium exchange with D_2_O. High resolution mass spectrometry experiments were performed with a dual electrospray interface (ESI) and a quadrupole time‐of‐flight mass spectrometer (Q‐TOF, Agilent 6530 Series Accurate‐Mass Quadrupole Time‐of‐Flight LC/MS, Agilent Technologies Italia S.p.A., Cernusco sul Naviglio, Italy). Full‐scan mass spectra were recorded in the mass/charge (m/z) range 50–3000 Da. Melting points (MP) for solid final compounds were determined by the capillary method on a Stuart Scientific SMP3 electrothermal apparatus and are uncorrected. RP‐HPLC analyses were performed on a system equipped with automatic injector and a Waters Breeze 1525 pump coupled with a Waters 2489 UV detector (Waters SpA, Sesto San Giovanni, Italy). The UV detection was measured at λ 254 and 370 nm. Clog P values of the data set were computed by using ChemDraw version 15.0 (PerkinElmer, Milan, Italy), ALOGPS 2.1 (VCCLAB, Virtual Computational Chemistry Laboratory, http://www.vcclab.org) and ChemSketch 2017 version 2.1 (ACD/ChemSketch, Advanced Chemistry Development, Inc., Toronto, ON, Canada, www.acdlabs.com).

All compounds were synthesized following Snyder's procedures^[4]^ with slight modifications. Compounds **2**,^[19]^ **14**,^[20]^ **15** ^[21]^ and **19** ^[22]^ have already been described; their analytical data agreed with those reported in quoted references.

### Synthesis of compounds 1–12

A suspension of 4‐cyano or 4‐nitroaniline (2 mmol) in 10 mL 6 N HCl was heated until the solid is solubilized, then the mixture cooled to 0 °C. A solution of NaNO_2_ (0.14 g, 2 mmol) in 1 mL of water was added and the mixture left under stirring for 30 min. In the order, solutions of 2‐furaldehyde (0.19 g, 2 mmol) in 2 mL of acetone and CuCl_2_ (0.04 g, 0.3 mmol) in 1 mL of water were added and the mixture was kept under stirring for 5 h. The precipitate formed was filtered and washed with distilled water. This intermediate compound was then solubilized in DMF (2 mL) and slowly added to an aqueous solution of the appropriate amine/hydrazine/hydrazide derivative (1.8 mmol). A catalytic amount of 4 N HCl was added and the solution was left under stirring at room temperature for 24 h. The mixture was extracted with CHCl_3_ (3×15 mL), the organic phase abundantly washed with H_2_O to remove the DMF and dried with Na_2_SO_4_. The solvent was evaporated under reduced pressure to give the crude product that was purified by column chromatography with hexane/ethyl acetate: 6/4 or 7/3 v/v as the mobile phase.

***N*** **′‐((5‐(4‐nitrophenyl)furan‐2‐yl)methylene)thiophene‐2‐carbohydrazide** **(1)**. Yellow crystals; yield: 25 %. ^1^H NMR (300 MHz, DMSO‐*d_6_*) *δ* 7.14 (d, *J*=3.5 Hz, 1H, furan), 7.23 (t, *J*=3.5 Hz, 1H, thiophene), 7.47 (d, *J*=3.5, 1H, furan), 7.89–8.40 (m, 7H arom.), 12.20 (brs, 1H, NH). ESI‐MS (C_16_H_10_N_3_O_4_S, [M−H]^−^) calcd. *m/z*=340.0390, found: 340.0392. MP 241–243 °C.

***N*** **‐(4‐methylpiperazin‐1‐yl)‐1‐(5‐(4‐nitrophenyl)furan‐2‐yl)methanimine** **(3)**. Red crystals; yield: 50 %. ^1^H NMR (300 MHz, DMSO‐*d_6_*) *δ* 2.21 (s, 3H, CH_3_), 2.41–2.50 (m, 4H, piperazine), 3.13 (t, *J*=5.2 Hz, 4H, piperazine), 6.70 (d, *J*=4.1 Hz, 1H, furan) 7.35 (d, *J*=4.1 Hz, 1H, furan), 7.54 (s, 1H, aldimine), 7.91 (d, *J*=8.5 Hz, 2H, ArNO_2_), 8.26 (d, *J*=8.5 Hz, 2H, ArNO_2_). ESI‐MS (C_16_H_19_N_4_O_3_, [M+H]^+^) calcd. *m/z*=315.1453, found 315.1456. MP 122–124 °C.

***N*** **′‐((5‐(4‐cyanophenyl)furan‐2‐yl)methylene)thiophene‐2‐carbohydrazide** **(4)**. Yellow crystals; yield: 20 %. ^1^H NMR (300 MHz, DMSO‐*d_6_*) *δ* 7.11 (d, *J*=3.5 Hz, 1H, furan), 7.23 (t, *J*=4.7 Hz, 1H, thiophene), 7.40 (d, *J=*3.5 Hz, 1H, furan), 7.80–8.05 (m, 6H, arom.), 8.37 (s, 1H, aldimine), 11.86 (brs, 1H, NH). ESI‐MS (C_17_H_12_N_3_O_2_, [M+H]^+^) calcd. *m/z*=322.0648, found 322.0643. MP 245 °C (dec).

***N*** **′‐((5‐(4‐cyanophenyl)furan‐yl)methylene)isonicotinohydrazide** **(5)**. Yellow crystals; yield: 30 %. ^1^H NMR (300 MHz, DMSO‐*d_6_*) *δ* 7.14 (d, *J*=3.5 Hz, 1H, furan), 7.40 (d, *J*=3.5 Hz, 1H, furan), 7.80 (d, *J*=5.8 Hz, 2H, pyridine), 7.91 (d, *J*=8.5 Hz, 2H, ArCN), 7.96 (d, *J*=8.5 Hz, 2H, ArCN), 8.03 (brs, 1H, NH), 8.40 (s, 1H, aldimine), 8.75 (d, *J*=5.8 Hz, 2H, pyridine). ESI‐MS (C_18_H_11_N_4_O_2_, [M−H]^−^) calcd. *m/z*=315.0880, found 315.0879. MP 240 °C (dec).

***N*** **′‐((5‐(4‐cyanophenyl)furan‐2‐yl)methylene)acetohydrazide** **(6)**. Orange crystal; yield: 35 %. ^1^H NMR (300 MHz, DMSO‐*d_6_*) *δ* 2.18 (s, 3H, CH_3_), 7.00 (d, *J*=3.6 Hz, 1H, furan), 7.35 (d, *J*=3.6 Hz, 1H, furan), 7.87–7.94 (m, 4H, arom.), 8.07 (s, 1H, aldimine), 11.33 (brs, 1H, NH). ESI‐MS (C_14_H_11_N_3_O_2_, [M+Na]^+^) calcd. *m/z*=276.0747, found 276.0743. MP 190 °C (dec).

***N*** **′‐((5‐(4‐cyanophenyl)furan‐2‐yl)methylene)‐2‐phenyl‐acetohydrazide** **(8)**. Orange crystals; yield: 35 %. ^1^H NMR (300 MHz, DMSO‐*d_6_*) *δ* 3.99 (s, 2H, CH_2_), 7.05 (d, *J*=3.5 Hz, 1H, furan), 7.29–7.40 (m, 6H, arom.), 7.88–7.97 (m, 4H, arom.), 8.15 (s, 1H, aldimine), 11.45 (brs, 1H, NH). ESI‐MS (C_20_H_16_N_3_O_2_, [M+H]^+^) calcd. *m/z*=330.1239, found 330.1236. MP 197 °C (dec).

**(4‐(5‐(((2‐chloro‐4‐hydroxyphenyl)imino)methyl)furan‐2‐yl)benzonitrile** **(9)**. Yellow crystals; yield: 25 %. ^1^H NMR (500 MHz, DMSO‐*d_6_*) *δ* 6.76 (dd, *J=*8.5 Hz, 1H, arom.), 6.90 (d, *J*=2.9 Hz, 1H, arom.), 7.22 (d. *J*=8.8 Hz, 1H, arom.), 7.28 (d, *J*=3.6 Hz, 1H, furan), 7.45 (d, *J*=3.6 Hz, 1H, furan), 7.93 (d, *J*=8.5 Hz, 2H, arom.), 7.99 (d, *J*=8.5 Hz, 2H, arom.), 8.41 (s, 1H, aldimine), 9.95 (brs, 1H, OH). ESI‐MS (C_18_H_10_ClN_2_O_2_, [M−H]^−^) calcd. *m/z*=321.0429, found 321.0432. MP 196–198 °C.

**4‐((5‐(2‐(4‐methoxyphenyl)hydrazin‐1‐ylidene)methyl)furan‐2‐yl))benzonitrile** **(10)**. Orange crystals; yield: 40 %; ^1^H NMR (300 MHz, DMSO‐*d_6_*) *δ* 3.68 (s. 3H, OCH_3_), 6.76 (d, *J*=4.1 Hz, 1H, furan), 6.84 (d, *J*=9.9 Hz, 2H, arom.), 7.00 (d, *J*=9.9 Hz, 2H, arom.), 7.30 (d, *J*=3.5 Hz, 1H, furan), 7.72 (s, 1H, aldimine) 7.84–7.89 (m, 4H, arom.), 10.36 (brs, 1H, NH). ESI‐MS (C_19_H_14_N_3_O_2_, [M−H]^−^) calcd. *m/z*=316.1083, found 316.1099. MP 138–140 °C.

**4‐(5‐((2‐(4‐(methylsulfonyl)phenyl)hydrazono)methyl)furan‐2‐yl)benzonitrile (11)**. Dark‐orange crystals; yield: 25 %; ^1^H NMR (300 MHz, DMSO‐*d_6_*) *δ* 3.10 (s. 3H, CH_3_), 6.95 (d, *J*=4.1 Hz, 1H, furan), 7.19 (d, *J*=8.7 Hz, 2H, arom.), 7.35 (d, *J*=3.5 Hz, 1H, furan), 7.73 (d, *J*=8.7 Hz, 2H, arom.), 7.72 (s, 1H, aldimine), 7.87–7.94 (m, 4H, arom.), 11.12 (brs, 1H, NH). ESI‐MS (C_19_H_16_N_3_O_3_S, [M+H]^+^) calcd. *m/z*=366.0909, found 366.0906. MP 175 °C (dec).

**((5‐(4‐cyanophenyl)furan‐2‐yl))methylidene)hydrazin‐1‐yl)benzene‐1‐sulfonamide** **(12)**. Dark‐orange crystals; yield: 32 %; ^1^H NMR (300 MHz, DMSO‐*d_6_*) *δ* 6.92 (d, *J*=3.5 Hz, 1H, furan), 7.08 (brs, 2H, NH_2_), 7.13 (d, *J*=8.2 Hz, 2H, arom.), 7.35 (d, *J*=3.5 Hz, 1H, furan), 7.67 (d, *J*=8.7 Hz, 2H, arom.), 7.84–7.96 (m, 5H, arom., aldimine), 10.91 (brs, 1H, NH). ESI‐MS (C_18_H_13_N_4_O_3_S, [M−H]^−^) calcd. *m/z*=365.0706, found 365.0699. MP 217–219 °C.

### Synthesis of compound 13

The procedure was the same as for compounds **1**–**12**, using 3‐methoxybenzaldehyde (0.27 g, 2.0 mmol) instead of 2‐furaldehyde, and 2‐thiophenecarboxylic acid hydrazide (0.43 g, 3.0 mmol). After evaporation of organic extract, the crude product obtained was purified by crystallization from absolute ethanol.

***N*** **′‐[(4′‐cyano‐2‐methoxy[1,1′‐biphenyl]‐4‐yl)methylidene]thiophene‐2‐carbohydrazide** **(13)**. White ivory crystals; yield: 24 %. ^1^H NMR (300 MHz, DMSO‐*d_6_*) *δ* 3.82 (s. 3H, OCH_3_), 7.01 (d, *J=*7.2 Hz, 1H, arom.), 7.19 (t, *J*=3.5 Hz, 1H, thiophene), 7.30–8.20 (m, 8H, arom.), 8.39 (s, 1H, aldimine), 11.86 (brs, 1H, NH). ESI‐MS (C_20_H_16_N_3_O_2_S, [M+H]^+^) calcd. *m/z*=362.0690, found 362.0699. MP 255 °C (dec).

### Synthesis of compounds 14–21

2.0 mmol of aldehyde (2‐furaldehyde for **14**–**18**; 3‐methoxybenzaldehyde for **19** and **20**; 3,4,5‐trimethoxybenzaldehyde for **21**) were solubilized in 5 mL of acetone and slowly added to the aqueous solution of the appropriate amine/hydrazine/hydrazide derivative, with a catalytic amount of HCl 4 N. The mixture was stirred at room temperature for 24 h, then extracted with ethyl acetate (3×15 mL) and the organic layer dried over anhydrous Na_2_SO_4_. Compound **21** was filtered after precipitation from the acetone/water mixture. The solvent was evaporated under reduced pressure to give the crude product that was purified by crystallization from absolute ethanol.

**1‐(Furan‐2‐yl)‐*N*‐(4‐methylpiperazin‐1‐yl)methanimine** **(16)**. Brown oil; yield: 38 %; ^1^H NMR (300 MHz, DMSO‐*d_6_*) *δ* 2.39 (s, 3H, CH_3_), 2.66 (t, *J*=3.1 Hz, 4H, 2xCH_2_), 3.24 (t, *J*=3.1 Hz, 4H, 2xCH_2_), 6.41 (t, *J*=1.5 Hz, 1H, furan), 6.45 (dd, *J*=1.5 Hz, 1H, furan), 7.26 (s, 1H, aldimine), 7.41 (d, *J*=2.0 Hz, 1H, furan). ESI‐MS (C_10_H_16_N_3_O, [M+H]^+^) calcd. *m/z*=194.1290, found 194.1286.

***N*** **′‐((furan‐2‐yl)methylidene)‐2‐phenylacetohydrazide** **(17)**. White ivory crystals; yield: 46 %. ^1^H NMR (300 MHz, DMSO‐*d_6_*) *δ* 3.50 (s, 2H, CH_2_), 6.50 (t, *J*=2.1 Hz, 1H, furan), 6.86 (d, *J*=2.0 Hz, 1H, furan), 7.18–7.30 (m, 5H, arom.), 7.80 (d, *J*=2.1 Hz, 1H, furan), 7.88 (s, 1H, aldimine), 11.30 (brs, 1H, NH). ESI‐MS (C_13_H_13_N_2_O_2_, [M+H]^+^) calcd. *m/z*=229.0974, found 229.0980. MP 156–160 °C.

**4‐(‐2‐((furan‐2‐yl)methylidene)hydrazinyl)benzene‐1‐sulfonamide** **(18)**. Orange crystals; yield: 47 %. ^1^H NMR (300 MHz, DMSO‐*d_6_*) *δ* 6.57 (t, *J*=1.6 Hz, 1H, furan), 6.72 (d, *J*=1.6 Hz, 1H, furan), 7.04 (brs, 2H, NH_2_), 7.05 (d, *J*=5.3 Hz, 2H, arom.), 7.63 (d, *J*=5.2 Hz, 2H, arom.), 7.71 (s, 1H, aldimine), 7.81 (d, *J*=1.6 Hz, 1H, furan), 10.72 (brs, 1H, NH). ESI‐MS (C_11_H_12_N_3_O_3_S, [M+H]^+^) calcd. *m/z*=266.0597, found 266.0593. MP 161–165 °C.

**4‐(2‐((3‐methoxyphenyl)methylidene)hydrazinyl)benzene‐1‐sulfonamide** **(20)**. Orange crystals; yield: 25 %; ^1^H NMR (300 MHz, DMSO‐*d_6_*) *δ* 3.79 (s, 3H), 6.89 (d, *J*=4.1 Hz, 1H, arom.), 7.07 (brs, 2H, NH_2_), 7.14 (d, *J*=8.8 Hz, 2H, arom.), 7.21–7.34 (m, 3H, arom.), 7.65 (d, *J*=8.9 Hz, 2H, arom.), 7.90 (s, 1H, aldimine), 10.79 (brs, 1H, NH). ESI‐MS (C_14_H_14_N_3_O_3_S, [M−H]^−^) calcd. *m/z*=304.0753, found 304.0756. MP 214–218 °C.

**1‐(((3,4,5‐trimethoxyphenyl)methylidene)amino)imidazolidine‐2,4‐dione (21)**. White solid; yield: 70 %. ^1^H NMR (300 MHz, DMSO‐*d_6_*) *δ* 3.68 (s, 3H, OCH_3_), 3.80 (s, 6H, 2xOCH_3_), 4.31 (s, 2H, CH_2_), 7.01 (s, 2H, arom.), 7.73 (s, 1H, aldimine), 11.17 (brs, 1H, NH). ESI‐MSv(C_13_H_16_N_3_O_5_, [M+H]^+^) calcd. *m/z*=294.1086, found 294.1083. MP 270 °C (dec.).

### Chromatographic measures

Stability studies in buffered solution were performed following an already described procedure,^[23]^ on a Phenomenex C18 column (150×4.6 mm i.d., 3 μm particle size; Phenomenex, Castel Maggiore, Italy) using a mobile phase consisting of a mixture of methanol‐water (75 : 25 v/v, with aqueous formic acid 0.1 %). The used flow rate was 0.500 mL/min while the injection volume was 20 μL. Wavelength of UV‐Vis detector was adjusted at 254 nm. The chemical stability was evaluated in a phosphate buffer solution pH 7.4 (10 mM HPO_4_^2−^/H_2_PO_4_^−^; 100 mM NaCl) at 37 °C. Five different concentrations from 0.5 μM to 20 μM were studied in a 2 h time range (data not shown). Each concentration (0.5, 1, 5, 10 and 20 μM) was tested in triplicate starting from 3 different stocks solution (1 mM) prepared separately. The time range was extended to 6 h only for the samples at 20 μM concentration (Figure 6a).

Log *k*’ values (*k’*=(t_r_‐t_0_)/t_0_) and purity determinations were carried out using a Phenomenex Gemini C18 4.6×150 mm, with 3 μm size particles, built on a Waters double pump HPLC system in isocratic conditions. Injection volumes were 10 μL, flow rate was 0.5 mL/min, and detection was performed with UV (λ=254 and 370 nm). Samples were prepared by dissolving 0.1 mg/mL of the solute in 10 % v/v DMSO and 90 % v/v methanol. Retention times (t_r_) were measured at least from three separate injections, and dead time (t_0_) was the retention time of deuterated methanol. The mobile phase was filtered through a Supelco Nylon‐66 membrane 0.45 μm (Merck Life Science Srl, Milan, Italy) before use. For each reference compound, the average t_r_ of three consecutive injections of 10 μL of sample was used to calculate the log *k’* values. The eluent consisted of five different mixtures of methanol and PBS buffer 10 mM at pH 7.4, with methanol/buffer ratios ranging from 80 : 20 to 60 : 40 v/v.

### Biological assays

#### Enzyme inhibition

Inhibition assays of *h*ChEs and *h*MAOs (all from Sigma Aldrich) were performed by using already published protocols.^[24]^ All compounds were assayed at 10 μM concentration and, for those showing inhibition >60 %, IC_50_ values was calculated by testing seven concentrations in the range 30–0.01 μM. Briefly, the classical spectrophotometric Ellman's test (for ChEs) and the fluorimetric detection of 4‐hydroxyquinoline (for MAOs) were adapted to a plate reader procedure with 96‐well microtiter plates (Greiner Bio‐One GmbH, Frickenhausen, Germany). Readings were made with Infinite M1000 Pro plate reader (Tecan, Cernusco s.N., Italy) and statistical regressions with Prism software (GraphPad Prism version 5.00 for Windows, GraphPad Software, San Diego, CA, USA). Kinetics of MAO inhibition for compound **9** were calculated with four concentrations of inhibitor (0, 0.5, 0.8, 1.5 μM) and seven concentrations of kynuramine (from 10 to 250 μM), and data analysed by means of the “Enzyme kinetics” module of Prism.

#### Cell cultures

Reagents for cell cultures were purchased from Life Technologies (ThermoFisher Scientific, Waltham, MA, USA) unless otherwise stated. The human tumour cell lines of neuroblastoma SH‐SY5Y were obtained from the National Cancer Institute, Biological Testing Branch (Frederick, MD, USA) and were maintained in the logarithmic phase at 37 °C in a 5 % CO_2_ humidified air in RPMI 1640+Glutamax medium supplemented with 10 % fetal bovine serum, 1 % penicillin and streptomycin and 50 μg/mL gentamicin.

#### Measurement of reactive oxygen species levels: DCF‐DA assay

ROS production in SH‐SY5Y cell line was detected using Cellular Reactive Oxygen Species Detection Assay Kit ab186027 (Abcam, Cambridge, UK), as previously described.^[1]^ Briefly, cells were seeded into 384‐well flat bottom transparent polystyrene microtiter plates (Greiner) at a plating density of 12000 cells per well. After seeding, microtiter plates was incubated overnight at 37 °C, then added with phenelzine sulfate (positive control) or test compounds, and dopamine HCl (10 nM) as ROS inductor. After incubation and washing, DCF‐DA was added, the plate was incubated, washed again with PBS, and the fluorescence read every 15 min in a 60 min time interval at 535 nm (excitation at 485 nm) using the Tecan Infinite M1000 Pro plate reader.

#### Transport measurement

An already reported protocol,^[1]^ based on the inhibitor‐stop method,^[25]^ was used. The recombinant WT CAC protein was reconstituted into liposomes as described previously.[^1^, ^26^] Briefly, transport was started by adding [^3^H]‐carnitine to proteoliposomes and stopped by the addition of N‐ethylmaleimide. After removal of the external substrate, intraliposomal radioactivity was measured by a liquid scintillation counter (Perkin Elmer, Milan, Italy). EC_50_ values were calculated using AATBioquest EC_50_ calculator.^[27]^ Statistical analysis was performed by Student's *t*‐test, as indicated in figure legends. Values of *p*<0.05 were considered statistically significant. Data points were derived from the mean of three different experiments, as specified in the figure legends.

## Conflict of interest

The authors declare no conflict of interest.

## Supporting information

As a service to our authors and readers, this journal provides supporting information supplied by the authors. Such materials are peer reviewed and may be re‐organized for online delivery, but are not copy‐edited or typeset. Technical support issues arising from supporting information (other than missing files) should be addressed to the authors.

Supporting Information
