# Supplementary material for: Synthesis and Biological Evaluation of Dantrolene‐Like Hydrazide and Hydrazone Analogues as Multitarget Agents for Neurodegenerative Diseases
Source: ChemMedChem. 2021 Jun 22;16(18):2807–16. doi: 10.1002/cmdc.202100209 (PMC8518391; doi:10.1002/cmdc.202100209)
Supplement: Supplementary file 1 — Supporting Information [file CMDC-16-2807-s001.pdf]

ChemMedChem

Supporting Information

Synthesis and Biological Evaluation of Dantrolene-Like Hydrazide and Hydrazone Analogues as Multitarget Agents for Neurodegenerative Diseases

Isabella Bolognino[+], Nicola Giangregorio[+], Annamaria Tonazzi, Antón L. Martínez, Cosimo D. Altomare, María I. Loza, Sara Sablone, Saverio Cellamare, and Marco Catto*

SUPPORTING INFORMATION

Table S1. Calculated log P values of compounds **1-21** **Page 2**

Figure S1. Plots of pIC_{50} values of enzymatic activities vs.

a) calculated Clog P values; b) log *k*' values experimentally determined **Page 3**

Table S1. Calculated log P values of compounds **1-21**.

Entry	Clog P[a]	Entry	Clog P[a]
**DAN**	1.63<br>1.65<br>1.43±0.59	**11**	3.15<br>4.28<br>3.12±0.66
**1**	4.18<br>3.84<br>2.94±0.61	**12**	2.58<br>3.92<br>2.95±0.64
**2**	2.63<br>3.01<br>1.75±0.58	**13**	3.19<br>4.57<br>3.27±0.58
**3**	2.58<br>2.17<br>1.27±0.55	**14**	-0.21<br>-0.24<br>0.02±0.49
**4**	3.12<br>3.72<br>2.64±0.63	**15**	1.59<br>2.34<br>1.54±0.57
**5**	2.32<br>3.03<br>1.45±0.60	**16**	1.27<br>0.54<br>-0.13±0.39
**6**	1.88<br>2.73<br>1.88±0.64	**17**	1.89<br>2.42<br>2.55±0.58
**7**	1.32<br>2.02<br>1.13±0.61	**18**	1.03<br>2.07<br>1.84±0.58
**8**	3.43<br>3.98<br>3.65±0.64	**19**	1.69<br>1.97<br>1.36±0.36
**9**	3.95<br>4.87<br>3.08±0.60	**20**	2.09<br>2.63<br>3.13±0.58
**10**	4.14<br>4.67<br>4.09±0.64	**21**	-0.081<br>1.08<br>1.22±0.51

[a]) First value: ChemDraw 15.0; second value: ALOGPS 2.1; third value: ChemSketch 2017.

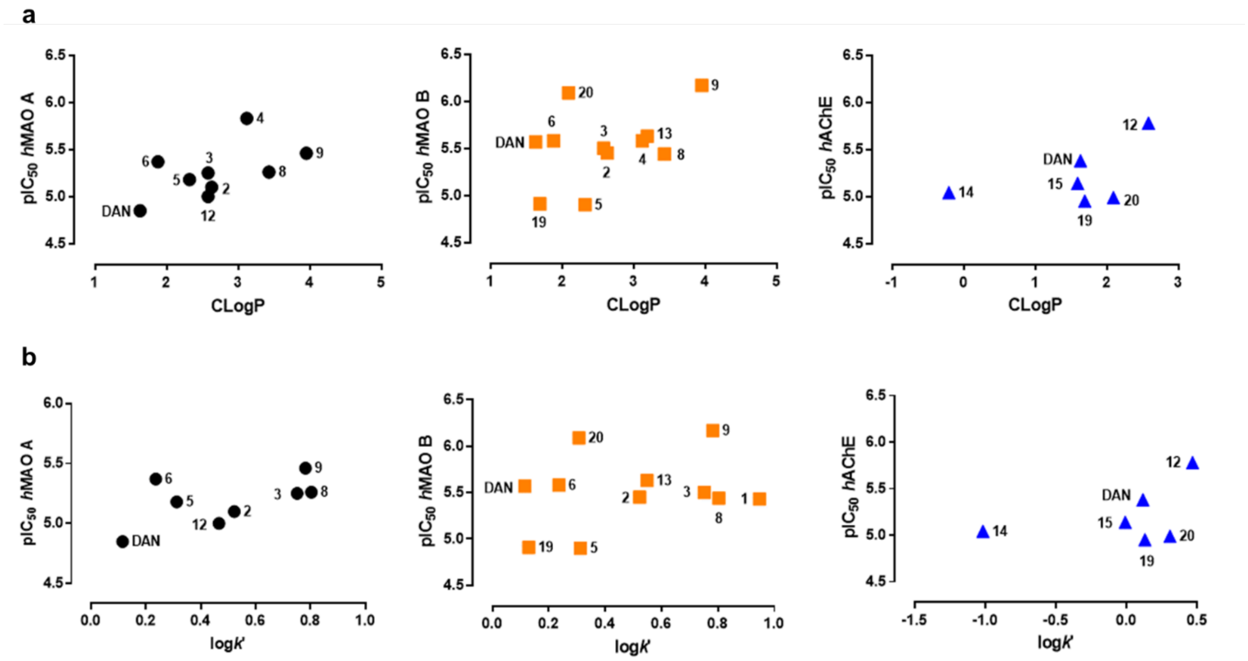

Figure S1. Plots of pIC_{50} values of enzymatic activities vs. a) calculated Clog P values (ChemDraw); b) log *k*' values experimentally determined (eluent mixture: MeOH/PBS buffer 60:40 v/v); only compounds with finite IC_{50} values are shown.
